# Atomic-layer soft plasma etching of MoS_2_

**DOI:** 10.1038/srep19945

**Published:** 2016-01-27

**Authors:** Shaoqing Xiao, Peng Xiao, Xuecheng Zhang, Dawei Yan, Xiaofeng Gu, Fang Qin, Zhenhua Ni, Zhao Jun Han, Kostya (Ken) Ostrikov

**Affiliations:** 1 Engineering Research Center of IoT Technology Applications (Ministry of Education), Department of Electronic Engineering, Jiangnan University, Wuxi 214122, China; 2 Analysis & Testing Center, Jiangnan University, Wuxi 214122, China; 3 Department of Physics, Southeast University, SEU Research Center of Converging Technology, Nanjing 211189, China; 4 CSIRO Manufacturing, P.O. Box 218, 36 Bradfield Road, Lindfield, New South Wales 2070, Australia; 5 School of Physics, The University of Sydney, Sydney, New South Wales 2006, Australia; 6 Institute for Future Environments and School of Chemistry, Physics and Mechanical Engineering, Queensland University of Technology, Brisbane, QLD 4000, Australia

## Abstract

Transition from multi-layer to monolayer and sub-monolayer thickness leads to the many exotic properties and distinctive applications of two-dimensional (2D) MoS_2_. This transition requires atomic-layer-precision thinning of bulk MoS_2_ without damaging the remaining layers, which presently remains elusive. Here we report a soft, selective and high-throughput atomic-layer-precision etching of MoS_2_ in SF_6_ + N_2_ plasmas with low-energy (<0.4 eV) electrons and minimized ion-bombardment-related damage. Equal numbers of MoS_2_ layers are removed uniformly across domains with vastly different initial thickness, without affecting the underlying SiO_2_ substrate and the remaining MoS_2_ layers. The etching rates can be tuned to achieve complete MoS_2_ removal and any desired number of MoS_2_ layers including monolayer. Layer-dependent vibrational and photoluminescence spectra of the etched MoS_2_ are also demonstrated. This soft plasma etching technique is versatile, scalable, compatible with the semiconductor manufacturing processes, and may be applicable for a broader range of 2D materials and intended device applications.

The electronic, mechanical, optoelectronic and catalytic properties of two-dimensional (2D) transition metal dichalcogenides (TMDs) such as MoS_2_ critically depend on the number of atomic stacking layers^1^,^2^,^3^,^4^,^5^,^6^,^7^,^8^,^9^,^10^. MoS_2_ has attracted rapidly growing attention from both academia and industry owing to its atomic-layer-dependent properties. Indeed, the electronic bandgap of MoS_2_ increases and transits from indirect to direct as the number of stacking layers decreases from multiple layers to monolayer^11^,^12^,^13^. Phase transition from semiconducting 2H to metallic 1T phases may also occur when the thickness is reduced to monolayer^14^,^15^. As such, atomically-thin MoS_2_ is a promising material for applications in valleytronic and optoelectronic devices, such as photodetectors, photovoltaics, and light emitters^11^,^16^,^17^; while multilayer MoS_2_ is suited for electronic and energy storage devices owing to its tunable bandgaps and loosely bound 2D atomic layers^18^,^19^,^20^.

However, exfoliated or synthesized MoS_2_ films usually contain non-uniform domains with different number of layers^21^. Post-growth thinning by methods of thermal annealing, laser and plasma etching has been actively pursued, but with only limited success^20^,^21^,^22^,^23^. For instance, thermal annealing has been applied to thin few-layer MoS_2_ flakes; yet the process was very time- and energy-consuming^22^,^23^. The thinned MoS_2_ films also possibly formed MoO_3_ due to high-temperature oxidation^23^ and showed non-uniform thickness across the surface and shrank laterally due to the uneven thermal sublimation effect^22^. Laser heating was also demonstrated to reduce the MoS_2_ thickness through thermal sublimation induced by light absorption^21^. However, monolayer domains were only achieved with careful control of the uniformity and dose of the laser exposure, which is challenging for scale-up^8^,^21^. In addition, argon-based plasmas facilitated layer-by-layer MoS_2_ thinning^20^, but may leave unwanted residues on the etched surface due to energetic Ar^+^ ion bombardment. It is therefore highly desired to develop a MoS_2_ thinning process with time- and energy-efficiency, ease to control and scale-up, and non-destruction to the remaining layers and substrate.

Here we report a soft, selective, high-throughput and uniform large-area plasma etching of MoS_2_. Compared to conventional plasma processes where energetic ions cause unwanted damage during etching, the present approach is based on reactive plasmas where the ion impact is intentionally reduced by generating the majority of the electrons with the energies insufficient for the effective ion generation or causing damage to the remaining MoS_2_ layers. The reactive radicals generated in such plasmas uniformly remove equal numbers of MoS_2_ layers irrespective of the initial thickness, without affecting the underlying SiO_2_ substrate and the exposed MoS_2_ layers. The etching rates can be tuned to achieve complete MoS_2_ removal and any desired number of atomic MoS_2_ layers including monolayer, starting from the pristine MoS_2_ domains of virtually any thickness. Layer-dependent vibrational and photoluminescence spectra of the etched MoS_2_ are also demonstrated. The process is fast, reproducible, and compatible with the established semiconductor microfabrication technologies, thus holding great promise to enable next-generation TMD-based devices.

## Results

In contrast to the traditional plasma etching processes, the soft plasma etching relies on SF_6_+N_2_ precursors dissociated in low-power, low-density radio-frequency plasma source (Supplementary Figure S1). Energetic ions can be minimized in such plasmas to significantly reduce structural damage to the 2D films. Specifically, the etching reactions used in this process are:


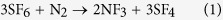



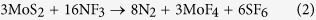



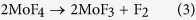


The key features of this plasma-based process include: (i) strong oxidant NF_3_ from reaction (1) can simultaneously react with both Mo and S; (ii) by-products MoF_4_, MoF_3_, F_2_ and SF_4_ of reactions (1–3) are volatile and are removed without residues on the etched surface, although some of these by-products are not so environment-friendly; (iii) the precursors and reaction by-products have negligible etching effect on the SiO_2_/Si substrate so that a highly-selective etching between MoS_2_ and SiO_2_ can be achieved; (iv) the rates of the soft etching can be effectively controlled by the plasma power density to enable *fine* (~0.8 mW/cm^3^) and *fast* (~1.2 mW/cm^3^) etching modes where any pre-determined number of MoS_2_ layers can be removed; (v) same number of MoS_2_ layers is removed uniformly across the domains with very different original number of layers. The soft etching mode is effective below the critical value (~1.5 mW/cm^3^) of the input power density. Beyond this critical value, structural damages to MoS_2_ emerge, *i.e.*, harsh etching mode. More details are provided below.

**Figure 1:**
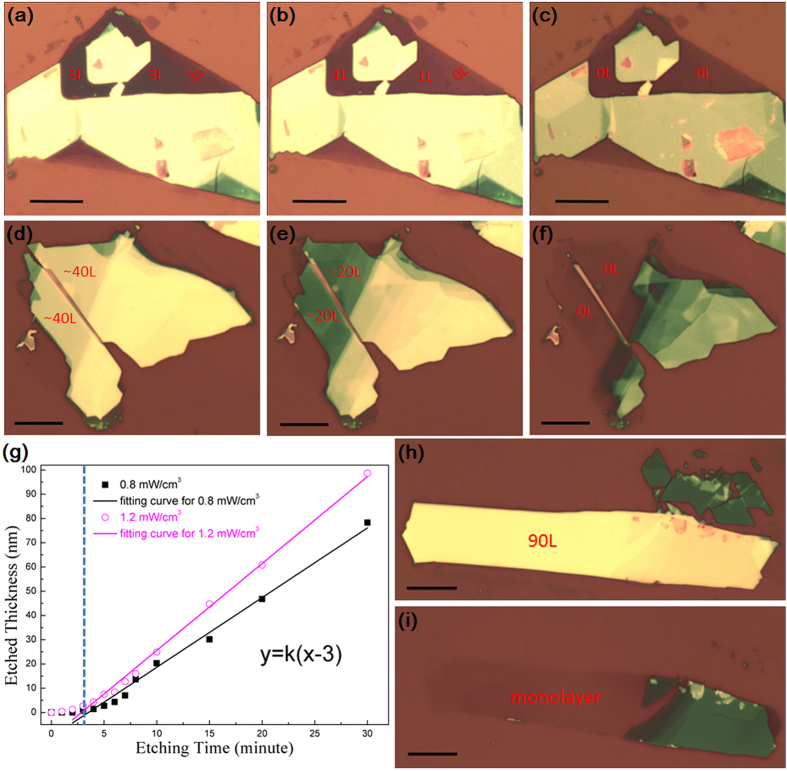
Plasma-etched MoS_2_ flakes at different input power densities. (**a**) Pristine MoS_2_ flake showing 1 and 3 layers; (**b**) MoS_2_-free (0L) and monolayer (1L) after 4 min *fine* plasma etching at 0.8 mW/cm^3^; and (**c**) MoS_2_-free surface after another 3 min *fine* etching at 0.8 mW/cm^3^. (**d**) Pristine MoS_2_ flake with ~40 layers; (**e**) ~20 layers after 8 min *fast* plasma etching at 1.2 mW/cm^3^; (**f**) MoS_2_-free surface after another 8 min *fast* etching at 1.2 mW/cm^3^. (**g**) The etched thickness as a function of time at 0.8 and 1.2 mW/cm^3^. (**h**) Large-area pristine MoS_2_ flake with ~90 layers; (**i**) MoS_2_ monolayer etched from the sample (**h**) using a combination of *fast* (first) and *fine* etching processes. All the scale bars in (**a**–**i**) are 10 μm.

Figure 1a–c show the etching result of a 4-layer MoS_2_ flake at an input power of 0.8 mW cm^−3^. The number of layers of pristine MoS_2_ flake was determined by optical contrast and atomic force microscopy (AFM), by assuming 0.7 nm as the thickness of a single S-Mo-S layer. From these images, it is found that 2 layers (~1.4 nm) and 1 layer (~0.7 nm) of MoS_2_ were successfully removed in two consecutive steps of 4 and 3 min, respectively. For comparison, thermal annealing in vacuum required 1 hr to remove 1 layer of MoS_2_^22^. Figure 1d–f show the etching result of a thick MoS_2 _flake starting with ~40 layers at an input power density of 1.2 mW cm^−3^. ~20 layers (~15 nm) of the MoS_2_ flake were removed in each step with the duration of 8 min, demonstrating the fast rate and high efficiency of this technique.

Remarkably, the present plasma thinning method had negligible etching effect to the SiO_2_/Si substrate. In a control experiment, we have covered half area of the SiO_2_/Si substrate by a shadow mask and treated it under the same SF_6_+N_2_ plasma conditions at either 0.8 or 1.2 mW cm^−3^ for 2 hr. No obvious boundaries or steps between the treated and the covered areas could be found from both AFM and optical images (Supplementary Figure S2), suggesting that the SF_6_+N_2_ plasma chemistry is highly selective to MoS_2_. As the SiO_2_/Si substrate is generally used as a reference in the AFM measurements to calculate the number of MoS_2_ layers, this allowed us to precisely determine the etching rate at different input power densities.

Figure 1g plots the etched thickness as a function of etching time at the power densities of 0.8 and 1.2 mW/cm^3^. Interestingly, the etching rates were low in the first 2–3 min in both cases. This can be explained by considering that reaction (1) requires certain time for the plasma to dissociate the SF_6_ molecules and produce enough NF_3_ radicals to etch MoS_2_, particularly at the beginning of the plasma discharge. The etching rates became stable after 3 min, as demonstrated by the linear fitting curves in Fig. 1g. The etching rates were about 2.8 and 3.6 nm/min for the power densities of 0.8 and 1.2 mW/cm^3^, respectively. Therefore, the former is referred as the fine etching mode because the low etching rate is best suited for high-precision removal of MoS_2_ atomic layers. On the other hand, the latter case is referred to as the fast etching mode which has higher etching rates and is more efficient for etching thicker MoS_2_ films.

By combining the fine and fast etching modes, domains of the arbitrary number of layers of MoS_2_ can be uniformly thinned across the sample. As shown in Fig. 1h,i and S3, a pristine MoS_2_ flake with 90 layers (~63 nm) and a size of ~60 μm was firstly subjected to the fast etching mode at 1.2 mW/cm^3 ^for 20 min. This led to a thinned MoS_2_ flake with only 3 layers (~2.4 nm). Then, the MoS_2_ flake was further etched under the fine mode for another 4 min, resulting in the formation of monolayer MoS_2_. The height of the MoS_2_ monolayer (~1.0 nm) was slightly larger compared to the monolayer MoS_2_ obtained by mechanical exfoliation^24^, which can be attributed to surface corrugation or the presence of adsorbed or trapped molecules^13^.

Importantly, the surface roughness of etched samples remained unchanged as compared to the pristine samples (Supplementary Figure S3), suggesting that the plasma etching process is indeed soft with the minimum damage induced. The layers that were left underneath after the etching were homogeneous. This is in stark contrast to the laser thinning method where ~3 times larger surface roughness was observed, possibly due to the different reaction rates of S and Mo atoms and/or the unremoved MoS_2_ traces on the surface^21^. The smooth surface obtained in the present soft plasma etching processes can be largely attributed to the NF_3 _radicals which etch both Mo and S uniformly to form volatile by-products. In addition, the domain sizes of the MoS_2_ flakes were not noticeably affected throughout the whole process, as compared to the conventional thermal or laser-based methods which significantly reduce the surface area of MoS_2_ domains^21^,^22^.

**Figure 2:**
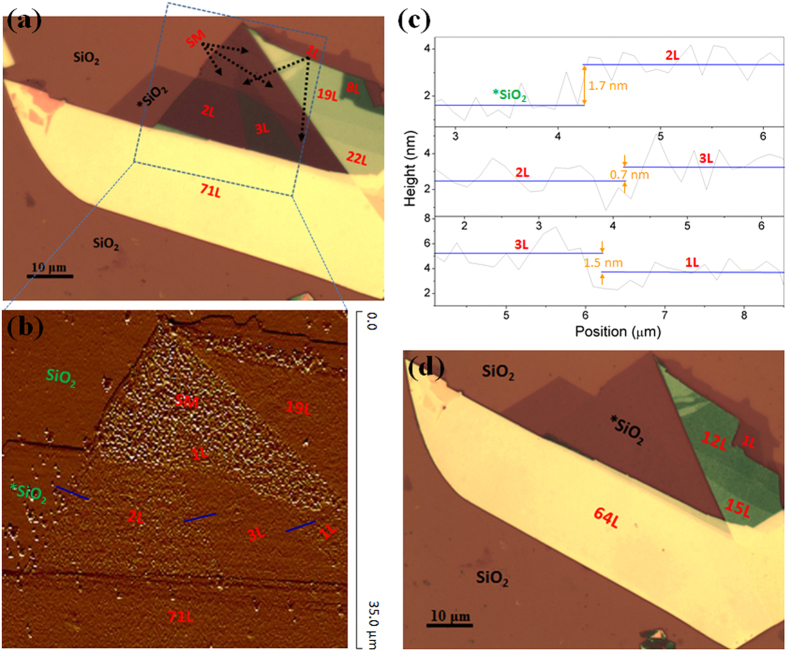
(**a**) The optical image of a representative etched sample consisting of flakes with the different number of layers including 1, 2, 3, 8, 19, 22, and 71 layers. SM denotes the MoS_2_ residues due to imcomplete removal of monolayer. (**b**) AFM image of the area squared in (**a**). (**c**) AFM depth profiles showing 1, 2 and 3 layers of MoS_2_ as marked by blue lines in (**b**). (**d**) The optical image of the same sample after another 7 min *fine* etching showing that 7 MoS_2_ layers have been removed uniformly from all domains starting with more than 7 layers.

Our plasma etching technique can also realize layer-by-layer thinning of MoS_2_ films with the domains of different number of layers. This aspect is particularly important because of the uneven thickness and patchy domains of the commonly produced MoS_2_ films. Figure 2a shows a representative etched sample consisting of sub-monolayer (SM), single, bi-, tri-, and multilayers, where the number of layers at distinct regions can be easily identified by both AFM and optical measurements (Fig. 2a–c). Notably, *SiO_2_ in the figure represents the exposed SiO_2_ surface after the complete removal of top MoS_2_ layers. It was also found that no residues were left for any layers with above 3 layers. However, for the incomplete removal of monolayer MoS_2_ that resulted in the SM area, a large number of MoS_2_ residues were identified (Fig. 2b). Scattered MoS_2_ residues were also found on 1-layer (1L) and 2-layer (2L) areas from Fig. 2b, although these residues were far less than that on the SM area. These observations suggested that the substrate had an impact in the etching process, particularly when the number of MoS_2_ layers is less than 3 layers. This substrate effect may be caused by the interactions between long-range van der Waals (vdW) and short-range polar and molecular forces^25^,^26^, which becomes negligible when the number of layers is large. Figure 2d shows the sample further subjected to a *fine* etching mode for another 7 min. Seven atomic layers were removed uniformly from the domains with more than 7 layers prior to etching, while only smooth SiO_2_ on the surface were found for domains with less than 7 layers. These results corroborate the large-area, uniform etching of MoS_2_ films using the soft plasma technique.

It should be noted that a height difference was observed between the bottom *SiO_2_ (0 layer areas) and the surrounding SiO_2_ (labeled as SiO_2_ in both optical and AFM images) in Figs 1b,c and 2a,d. This is because the pristine samples used for soft etching had been subjected to SF_6_+N_2_+H_2_ plasma treatment in the beginning at an input power density of 4 mW/cm^3^ for 20 min. Adding H_2_ into the plasma can effectively promote the etching of SiO_2_ and cleaning of substrate. About 20 nm SiO_2_ was etched after this step while no etching of MoS_2_ was found. The optical images of MoS_2_ sample before and after SF_6_+N_2_+H_2_ plasma treatment are shown in Figure S4. This observation demonstrated the easy control over selective etching between SiO_2_ and MoS_2_ by simply changing the plasma etchant gases. Details are out of scope of the present work but will be a subject of future studies.

**Figure 3:**
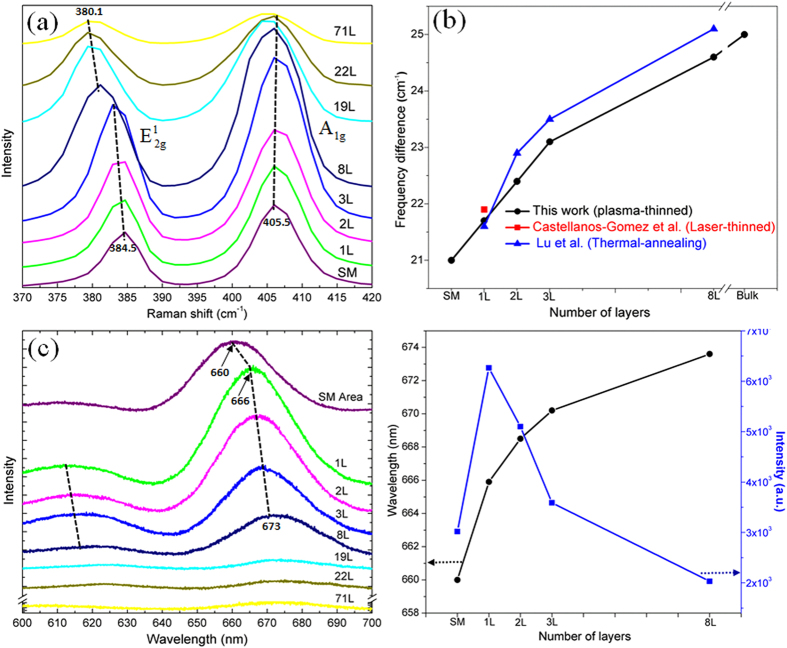
(**a**) Raman spectra of the etched MoS_2_ flakes with different thickness and MoS_2_ sub-monolayer. (**b**) The difference in Raman shifts between the 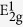 and A_1g_ peaks plotted as a function of the number of MoS_2_ layers. MoS_2_ layers thinned by the laser and thermal methods^21^,^22^ are also included for comparison. (**c**) PL spectra of the etched MoS_2_ flakes with different thickness and MoS_2_ sub-monolayer. (**d**) The wavelength and intensity of the prominent PL peak plotted as a function of the number of MoS_2_ layers.

The etched MoS_2_ is further characterized by Raman spectroscopy, which is a sensitive technique to determine the number of layers in ultrathin MoS_2_ flakes^24^,^27^,^28^,^29^. According to previous studies^24^,^28^, the peak positions of in-plane vibrational 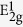 mode and out-of-plane vibrational A_1g_ mode vary monotonously with the number of MoS_2_ layers. Specifically, when the number of layers is decreased, A_1g_ shifts to lower frequencies due to weaker van der Waals forces which mostly affect the out-of-plane vibrations, while 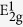 shifts to higher frequencies owing to the decreased dielectric screening of the long-range Coulombic interactions^22^,^30^,^31^. Figure 3a compares the Raman spectra obtained from different regions of an etched MoS_2_ sample (displayed in Fig. 2a), including SM area and numbers of 1, 2, 3, 8, 19, 22 and 71 layers. It is found that the A_1g_ peak shifted slightly to lower frequencies as the number of layers decreased, especially in the 1–8 layers range, while the 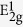 peak shifted up to higher frequencies. These Raman shifts are consistent with the predicted characteristics of thinned MoS_2_ layers.

Quantitatively, we also calculated the frequency difference between 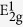 and A_1g_ in the plasma etched samples, as shown in Fig. 3b. The frequency difference decreased gradually from about 25 cm^−1^ for bulk film to 21.7 cm^−1^ for monolayer MoS_2_. This frequency shift is similar to the results obtained by other post-growth thinning methods, but the frequency difference value of the plasma-thinned monolayer or bilayer is larger than that of the pristine MoS_2_ produced by mechanical exfoliation^20^,^22^,^24^,^28^. Like other post-growth thinning methods such as laser thinning or chemical methods^13^,^21^, this discrepancy can be attributed to the presence of a small number of traces of MoS_2_ residues on the surface of the plasma-thinned monolayer or bilayer, as demonstrated in Fig. 2b. While this made the present etching method slightly inferior to Ar^+^ plasma method^20^ in thinning monolayer or bilayer, it is more efficient and credible in thinning thick layers. Interestingly, the frequency difference of SM area showed a further decrease as compared to that of monolayer MoS_2_, possibly due to the quantum confinement effect arising from the reduced lateral size of MoS_2_ residues.

In addition to the Raman vibrational fingerprints, ultrathin MoS_2_ layers also exhibit unique signatures in their PL spectra. Figure 3c shows the PL spectra of MoS_2_ with 1, 2, 3, 8, 19, 22 and 71 layers, as well as SM area. Two peaks located at ~670 and ~615 nm were identified in ultrathin layers (<8 layers), corresponding to the excitonic transitions between the minimum of the conduction band and the maxima of the two splitted valence bands, *i.e.*, the A_1_ and B_1_ excitons in MoS_2_^11^,^16^. In particular, the PL intensity increased gradually along with the decrease of the number of MoS_2_ layers, in a good agreement with previous reports^11^,^16^. Figure 3d shows the variations of peak position and intensity of the dominant PL peak as a function of the atomic layer numbers. As seen, the peak showed a blueshift from 673 to 666 nm as MoS_2_ was thinned to monolayer, suggesting the increased bandgap energy. The monolayer produced the highest PL intensity, which can be ascribed to the indirect to direct bandgap transition^8^,^16^. Interestingly, the SM area also exhibited a broad PL peak at ~660 nm with slightly reduced intensity as compared to that of monolayer MoS_2_^20^. Such observation may be attributed to the additional quantum confinement effect arising from the reduced lateral size and/or the distorted S-Mo-S sandwich structure in MoS_2_ residues.

It was noted that our soft plasma etching regime of MoS_2_ films was controlled by the input power density of the radio-frequency ICP source. The etching becomes harsh at a critical value of about 1.5 mW/cm^3^. Below this critical input power density, the MoS_2_ thinning process is dominated by radical reactions based on the SF_6_+N_2_ plasma chemistry. While at higher power densities, more high-energy electrons are generated, which enhances ion production and leads to uncontrollable damage to the samples through ion impact. In particular, at the input power density of 2 mW/cm^3^ the roughness of etched MoS_2_ samples increased significantly, with craters and pinholes clearly seen on the surface (Supplementary Figure S5).

To further explore the effect of the plasma electrons on the specific etching mode, we measured the electron energy distribution function (EEDF) using a Langmuir probe. The measurements were performed in a wide range of bias voltage, providing the I-V characteristic. The EEDF is generally defined as the second-order derivative of the measured I-V curve and can be expressed by the following equation^32^,^33^:


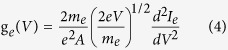


where A is the surface collection area of the Langmuir probe, e is the electron charge, and me is electron mass. The electron density ne and electron temperature Te can be derived from the measured EEDF by assuming a Maxwellian distribution^32^,^33^:


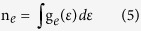



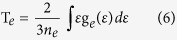


**Figure 4:**
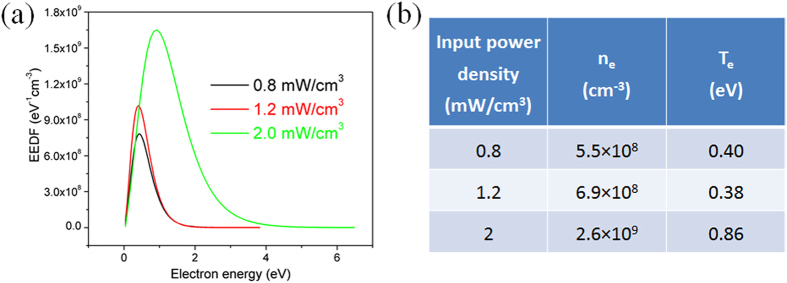
(**a**) The electron energy distribution function (EEDF) of the E-mode SF_6_+N_2_ discharges at three typical input power densities; (**b**) the corresponding calculated values of electron density (*n*_*e*_) and electron temperature (*T*_*e*_).

Figure 4a shows the experimental EEDF curves at the three input power densities of 0.8, 1.2 and 2 mW/cm^3^, corresponding to the fine and fast regimes of the soft etching mode, and the harsh etching mode, respectively. Figure 4b shows the values of n_e_ and T_e_ calculated based on Equations (5) and (6) respectively. It can be clearly seen that as long as the input power density was lower than the critical value of 1.5 mW/cm^3^, the electron temperature and density were below 0.4 eV and ~10^8 ^cm^−3^, respectively. These values were much lower compared to the conventional plasmas generated in the capacitively or inductively coupled configurations. As such, the fraction of electrons causing precursor ionization and dissociation was lower, so was the probability of ion-induced damage of underlying MoS_2_ layers. Furthermore, negligible high-energy electron tail (>2 eV) was observed in the EEDF, suggesting that electron-impact ionization and dissociation of the reactive radicals was not effective. Consequently, the effects of chemical reactions dominated over the ion bombardment in the MoS_2_ thinning process.

When the power density exceeded the critical value, the energy and density of electrons and ions increased significantly which may start to cause structural damage to the sample. The presence of higher-energy electrons (>2.0 eV) may also disrupt the S-Mo-S structure as the energy required to create vacancies in MoS_2_ is as low as 2.12 eV^34^. Another adverse effect is heat-induced evaporation, which may lead to surface defects at random positions. Nevertheless, as long as the input power density is lower than the critical value, the surface of multilayer MoS_2_ obtained by the soft plasma etching remains very smooth and the number of removed atomic layers is uniform across the whole sample surface. Furthermore, some encouraging results have been obtained on the atomic-layer soft etching of MoSe_2_ thin films (Figure S6), suggesting that this technique is also applicable to other TMDs. The comparative advantages of the present soft plasma etching techniques over other existing techniques are also summarized in Supplementary Table S1.

In summary, we have demonstrated a versatile and effective plasma technique for the soft, selective, and uniform layer-by-layer etching of MoS_2_ using SF_6_ + N_2_ precursors. Using the plasma power densities below the critical value of ~1.5 mW/cm^3^, equal number of MoS_2_ atomic layers can be removed from all the MoS_2_ areas irrespective of their original thickness. Large-area MoS_2_ flakes with arbitrary number of layers can be effectively thinned by combining the fine and fast etching modes switched-over by the power density of the plasma discharge. The plasma chemistry is highly-selective to MoS_2_ with negligible etching of the SiO_2_/Si substrate. The surface of etched MoS_2_ samples remained homogeneous and smooth with no shrinkage in the original domain sizes. The present approach is generic and may be used in the development of plasma-based etching processes of other TMDs pursued for applications in next-generation electronic, optoelectronic and other integrated devices and systems. It could also be promising for the catalytic applications of MoS_2_ since plasma treatment is one of the important techniques for generating active sites in MoS_2_ layers^35^. Furthermore, our results may contribute to the development of soft plasma etching processes for large-scale semiconductor microfabrication technologies.

## Methods

### MoS_2_ preparation and plasma thinning

Thick MoS_2_ layers were fabricated by mechanical exfoliation from bulk single-crystal MoS_2_ and deposited onto a Si/SiO_2_ (300 nm) substrate. These multilayers were characterized by a combination of optical microscopy, atomic force microscopy (AFM) and Raman spectroscopy. A planar low-frequency (0.5 MHz) inductively-coupled plasma (ICP) source was applied to etch the MoS_2_ multilayers without any external heating. The plasma was excited in the E-mode of ICP with the precursor gases of N_2_ and SF_6_ fed at the flow rates of 1.0 and 4.5 sccm, respectively. The E-mode discharge can be stably maintained at very low input power densities so that the ion density was too low to induce destructive ion bombardment onto the processed samples^36^. Whether the plasma etching is *soft* or *harsh* depended strongly on the input power density, with a critical value of ~1.5 mW/cm^3^. Therefore, we adopted two low input power densities at 0.8 and 1.2 mW/cm^3^ for the *fine* and *fast* etching processes, respectively. The schematics of experimental setup of the E-mode ICP source is presented in Figure S1.

### Characterization

The optical contrast images were obtained using a Leica 4200 Optical Microscopy. The Raman and photoluminescence (PL) spectra were recorded using a LabRAM HR Evolution Raman system with 532 nm laser excitation. The laser power at the sample was lower than 0.5 mW to avoid any laser-induced heating. To obtain the Raman images, an X-Y stage was used to move the sample with a 200 nm step, and the corresponding Raman spectrum was recorded at every point. AFM is carried out using a Bruker Dimension ICON system in the tapping mode.

## Additional Information

**How to cite this article**: Xiao, S. *et al.* Atomic-layer soft plasma etching of MoS_2_. *Sci. Rep.* **6**, 19945; doi: 10.1038/srep19945 (2016).

## Supplementary Material

Supplementary Information
