# Supplementary material for: Atomic-layer soft plasma etching of MoS2
Source: Sci Rep. 2016 Jan 27;6:19945. doi: 10.1038/srep19945 (PMC4728689; doi:10.1038/srep19945)
Supplement: Supplementary Information [file srep19945-s1.pdf]

Supplementary Information

Atomic-layer soft plasma etching of MoS_2

Shaoqing Xiao[*,1,‡] , Peng Xiao[1,‡], Xuecheng Zhang[1], Dawei Yan[1], Xiaofeng Gu[1], Fang Qin[2], Zhenhua Ni[*,3], Zhao Jun Han[4], and Kostya (Ken) Ostrikov[*,4,5,6]

[1] Engineering Research Center of IoT Technology Applications (Ministry of Education), Department of Electronic Engineering, Jiangnan University, Wuxi 214122, China.

[2]Analysis & Testing Center, Jiangnan University, Wuxi 214122, China

[3]Department of Physics, Southeast University, SEU Research Center of Converging Technology, Nanjing 211189, China

[4]CSIRO Manufacturing, P.O. Box 218, Lindfield, New South Wales 2070, Australia

[5]School of Physics, The University of Sydney, Sydney, New South Wales 2006, Australia

[6]Institute for Future Environments and School of Chemistry, Physics and Mechanical Engineering, Queensland University of Technology, Brisbane, QLD 4000, Australia

E-mail: *larring0078@hotmail.com; zhni@seu.edu.cn; kostya.ostrikov@csiro.au*

[‡]These authors contributed equally to this work.

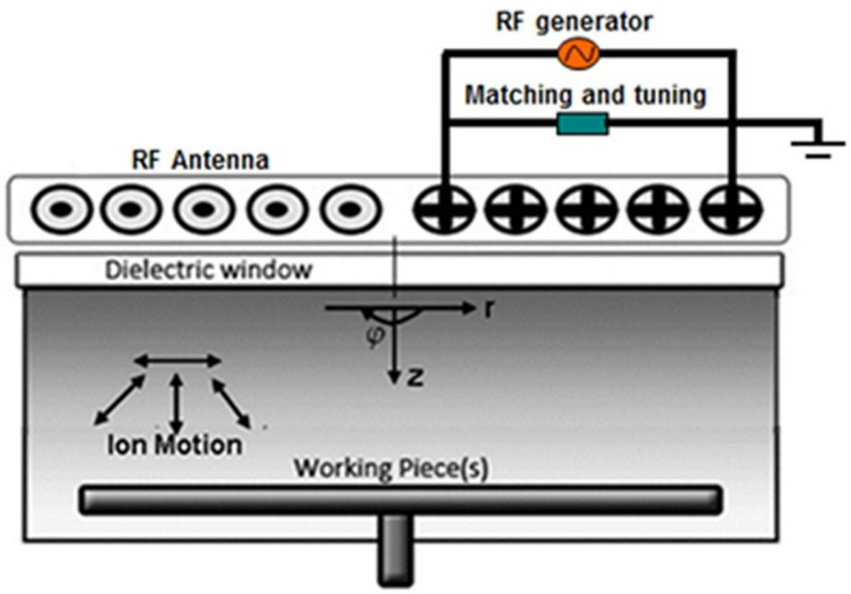

Figure S1 | The schematics of the experimental setup of the planar E-mode ICP source.

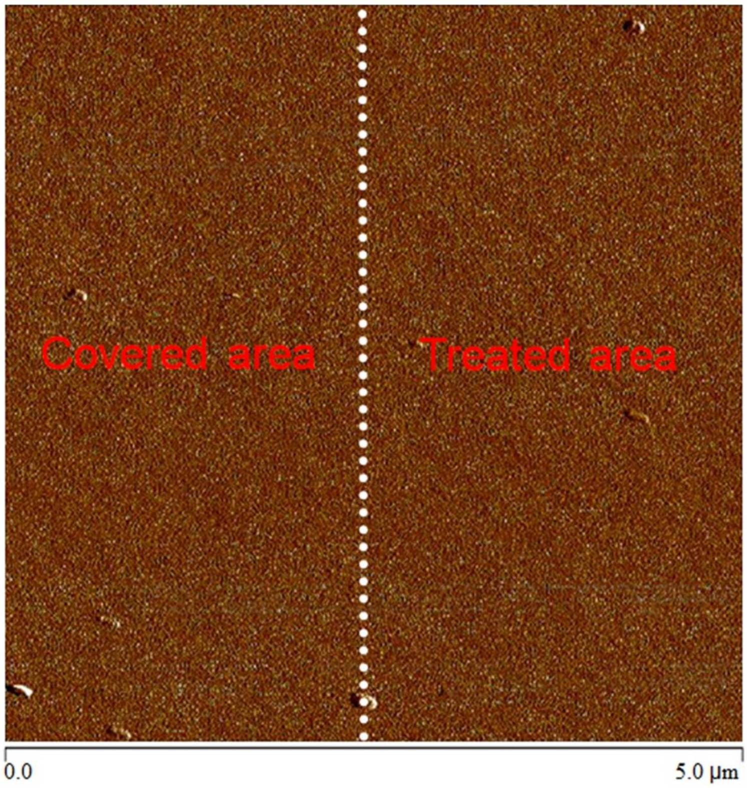

Figure S2 | A representative AFM surface morphology of the SiO_2/Si substrate consisting of the treated area under SF_6 + N_2 plasma environment for 2 hr and the covered area.

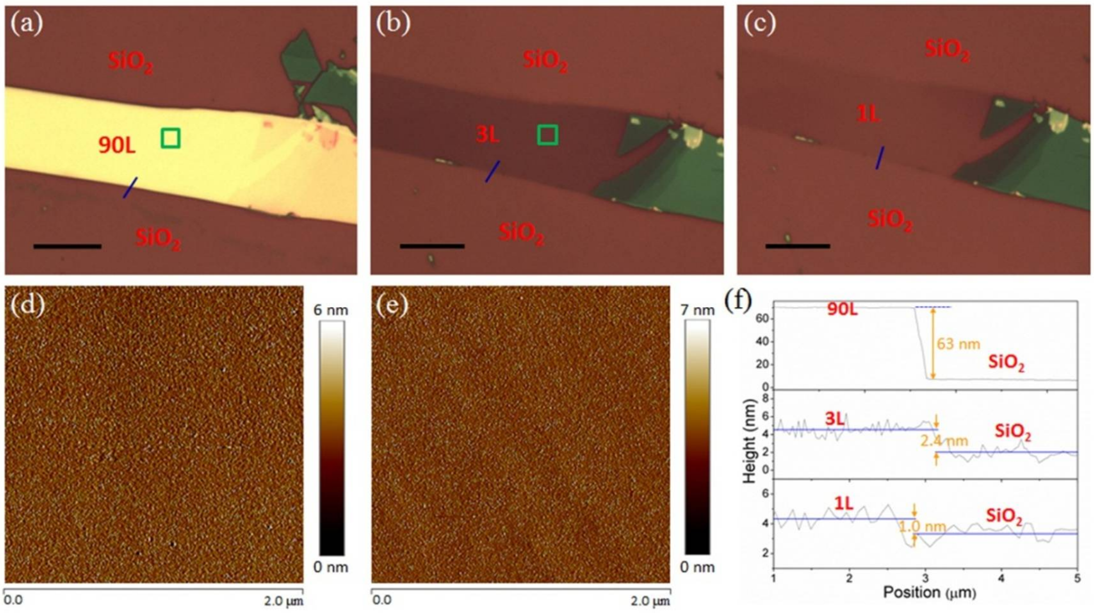

Figure S3 | (a) A large-area thick MoS_2 multilayer and (b,c) its corresponding etched samples by combining the fine and fast etching modes at power densities of 0.8 and 1.2 mW/cm^3, respectively. a) Optical image of pristine MoS_2 flakes; (b) after 20 min etching at 1.2 mW/cm^3; (c) after 4 min etching at 0.8 mW/cm^3. (d) and (e) are the corresponding AFM images of the squared regions in (a) and (b), respectively. The rms roughness in (d) and (e) is 0.7 nm and 0.8 nm, respectively. (f) The AFM depth profiles of MoS_2 as denoted by blue lines in (a-c). Scale bars in (a-c) are 10 μm.

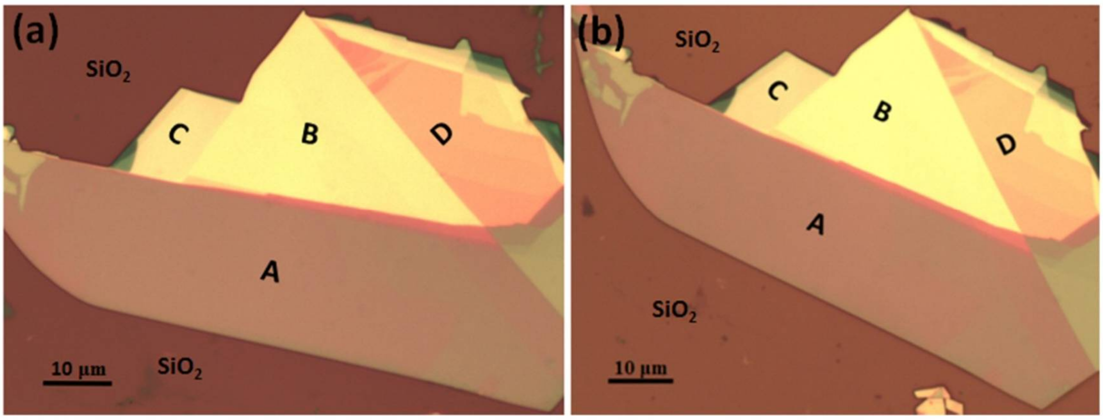

Figure S4 | Optical images of the pristine sample used for etching in Figure 2: before (a) and after (b) SF_6+N_2+H_2 plasma etching at an input power density of 4.0 mW/cm^3. The A area is made of ~158 MoS_2 layers, the B area ~87-91 layers, the C area ~53 layers, and the D area ~106-109 layers. The etching thickness of SiO_2 after this plasma process is estimated to be about 20 nm deduced from the AFM results.

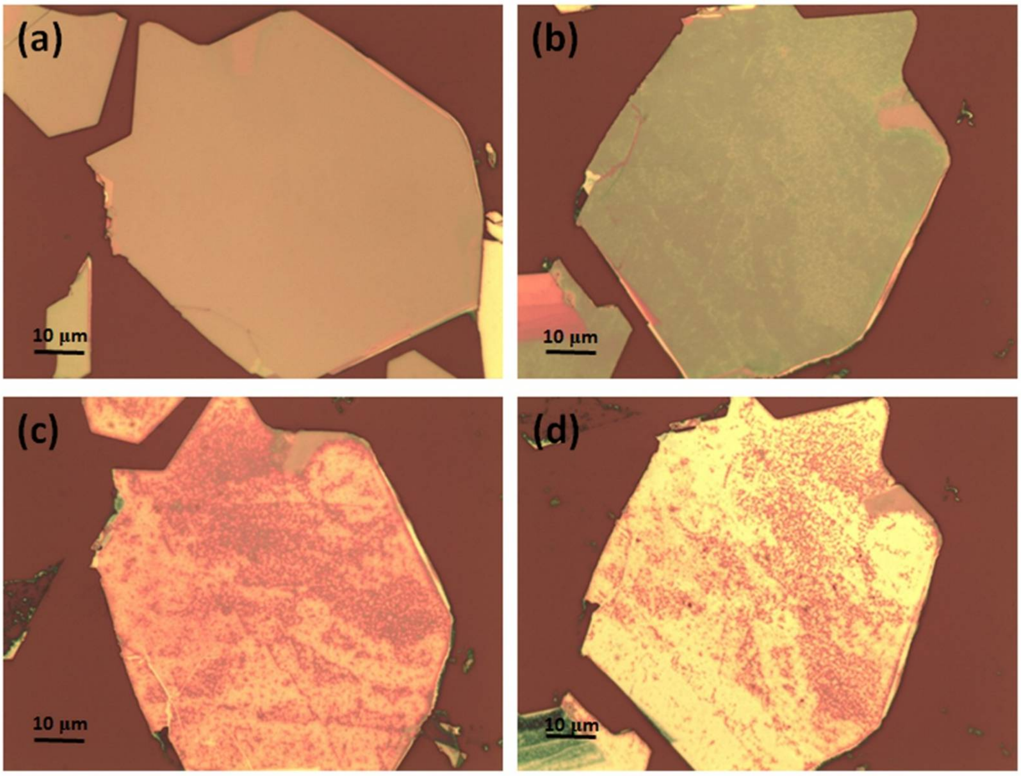

Figure S5 | (a) Optical images of a large-area thick MoS_2 flake. (b) The same sample after 10 min etching at a power density of 2 mW/cm^3; (c) after another 10 min etching at the same power; and (d) after further 10 min etching at the same power. Non-uniform and rough surface was observed on the etched MoS_2 flake.

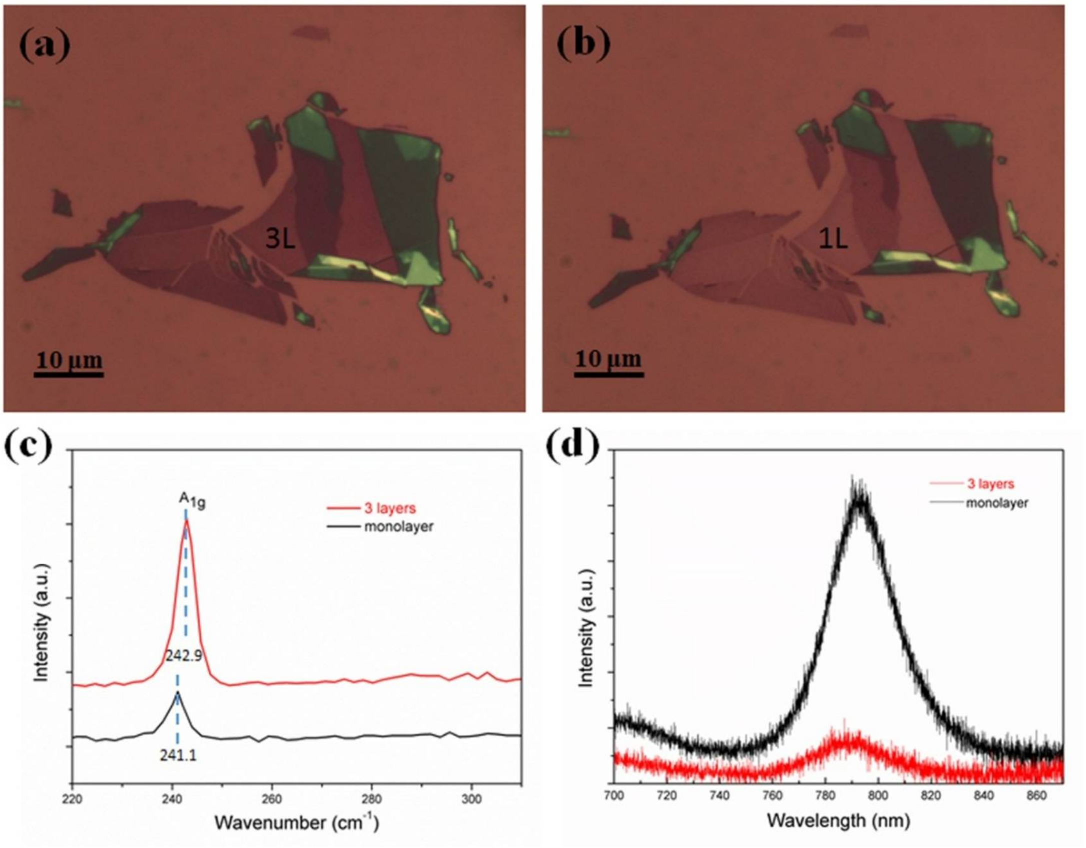

Figure S6 | Two typical optical images of plasma-etched $MoSe_2$ flakes. (a) Pristine $MoSe_2$ flake showing 3 layers; (b) monolayer (1L) after 1 min *fine* plasma etching; (c) corresponding Raman spectra and (d) PL spectra before and after this soft plasma thinning. About 2 layers were removed after the etching process. One can see that as the number of $MoSe_2$ layers was decreased, the A_{1g} Raman mode shifted from 242.9 to 241.1 cm^{-1}, which was in good agreement with previous report [see *Nano Lett. 12, 5576 (2012)*]. The PL spectra also showed a large enhancement when the 3 layers of $MoSe_2$ were thinned down to monolayer. Similar to MoS_2 PL results, this enhancement in PL for single-layer $MoSe_2$ can be attributed to an indirect-to-direct bandgap transition.

Supplementary Table

Table S1 | Summary of competitive advantages of our soft plasma etching method of thinning MoS_2 compared to other methods such as thermal annealing, laser thinning and Ar plasma thinning.

Features	Thermal thinning	Laser thinning	Ar Plasma thinning	**Soft etching**
Number of layers etched	1-7	Unkown	1-3	**Any***
Layers removal rate	1 layer/hr	Unkown	1 layer/115 s	**1 layer/180 s (fine etching mode) 290 layers/hr (fast etching mode)**
Domain area reduction	Yes	Yes	No	**No**
Etching residues left on surface	No	Yes	Yes	**No**
Damage to underlying layers	Yes	Yes	No	**No**
Selective etching between MoS_2 and SiO_2	Yes	Yes	Yes	**Yes**
Vacuum needed	Low	Low	Medium	**Medium**
Controllability on etching rate	Low	Unable	Medium	**High**
Scalability	Low	Low	High	**High**

*In this work the results of up to 90 layers are presented.
